# V-ATPase inhibition increases cancer cell stiffness and blocks membrane related Ras signaling - a new option for HCC therapy

**DOI:** 10.18632/oncotarget.14339

**Published:** 2016-12-28

**Authors:** Karin Bartel, Maria Winzi, Melanie Ulrich, Andreas Koeberle, Dirk Menche, Oliver Werz, Rolf Müller, Jochen Guck, Angelika M. Vollmar, Karin von Schwarzenberg

**Affiliations:** ^1^ Department of Pharmacy, Pharmaceutical Biology, Ludwig-Maximilians-University of Munich, 81377 Munich, Germany; ^2^ Biotechnology Center, Technische Universität Dresden, 01307 Dresden, Germany; ^3^ Chair of Pharmaceutical/Medicinal Chemistry, Institute of Pharmacy, Friedrich Schiller University Jena, 07743 Jena, Germany; ^4^ Kekulé Institute of Organic Chemistry and Biochemistry, University of Bonn, 53121 Bonn, Germany; ^5^ Department of Microbial Natural Products, Helmholtz Institute for Pharmaceutical Research Saarland (HIPS) - Helmholtz Centre for Infection Research (HZI), Saarland University, 66123 Saarbrücken, Germany

**Keywords:** HCC, cholesterol, V-ATPase, cell stiffness, Ras

## Abstract

Hepatocellular carcinoma (HCC) is the fifth most frequent cancer worldwide and the third leading cause of cancer-related death. However, therapy options are limited leaving an urgent need to develop new strategies. Currently, targeting cancer cell lipid and cholesterol metabolism is gaining interest especially regarding HCC. High cholesterol levels support proliferation, membrane-related mitogenic signaling and increase cell softness, leading to tumor progression, malignancy and invasive potential. However, effective ways to target cholesterol metabolism for cancer therapy are still missing. The V-ATPase inhibitor archazolid was recently shown to interfere with cholesterol metabolism. In our study, we report a novel therapeutic potential of V-ATPase inhibition in HCC by altering the mechanical phenotype of cancer cells leading to reduced proliferative signaling. Archazolid causes cellular depletion of free cholesterol leading to an increase in cell stiffness and membrane polarity of cancer cells, while hepatocytes remain unaffected. The altered membrane composition decreases membrane fluidity and leads to an inhibition of membrane-related Ras signaling resulting decreased proliferation *in vitro* and *in vivo*. V-ATPase inhibition represents a novel link between cell biophysical properties and proliferative signaling selectively in malignant HCC cells, providing the basis for an attractive and innovative strategy against HCC.

## INTRODUCTION

Hepatocellular carcinoma (HCC) is still one of the major causes of cancer-related death. Despite intensive research knowledge of the pathology remains poor resulting in a lack of therapy options. Hence it is urgent to elucidate new targets and strategies in treatment [1–4]. One promising approach that has come into focus lately is targeting cancer lipid and cholesterol metabolism pathways, which have been shown to be aberrant in cancer cells, especially in HCC [5, 6]. Statins, cholesterol synthesis inhibitors, were tested in several studies but until now with controversial outcome for anti-cancer therapy [7, 8]. Interestingly, the very potent V-ATPase inhibitor archazolid (arch) [9, 10] has recently been implicated in cholesterol regulation [11]. The V-ATPase is a proton pump which is involved in pH regulation and important for endocytotic pathways. Recently it has emerged as promising anti-cancer target as inhibition leads to apoptosis induction of in variety of cancer cells [12–15].

Cholesterol is of vital importance for cellular lipid bilayers and therefore for biophysical cell characteristics such as membrane stiffness, deformability and fluidity, but also for proliferation and signaling. Increasing evidence suggests that the loss of cell stiffness correlates with the malignancy and invasive potential supported by the observation that cancer cells are softer than their non-malignant counterparts [16, 17]. Importantly, membrane cholesterol is also essential for intracellular signaling as high cholesterol levels promote tumor progression as well as drug resistance [5, 18]. In membranes cholesterol is tightly packed into highly ordered lipid-rafts, together with saturated fatty acids and sphingolipids and plays an important role in signaling processes, membrane trafficking, motility and endocytosis [19].

The present study provides evidence that interfering with cholesterol metabolism by V-ATPase inhibition leads to an increased stiffness of tumor cells and affects membrane-related Ras signaling known to often be aberrant in malignant cells [20]. Such a link in biophysical and cell-biological principles in search of potent anti-tumor agents might lead to novel therapeutic options for treatment of HCC.

## RESULTS

### Archazolid A induces cancer cell stiffening due to alterations in membrane fluidity and polarity

**Figure 1:**
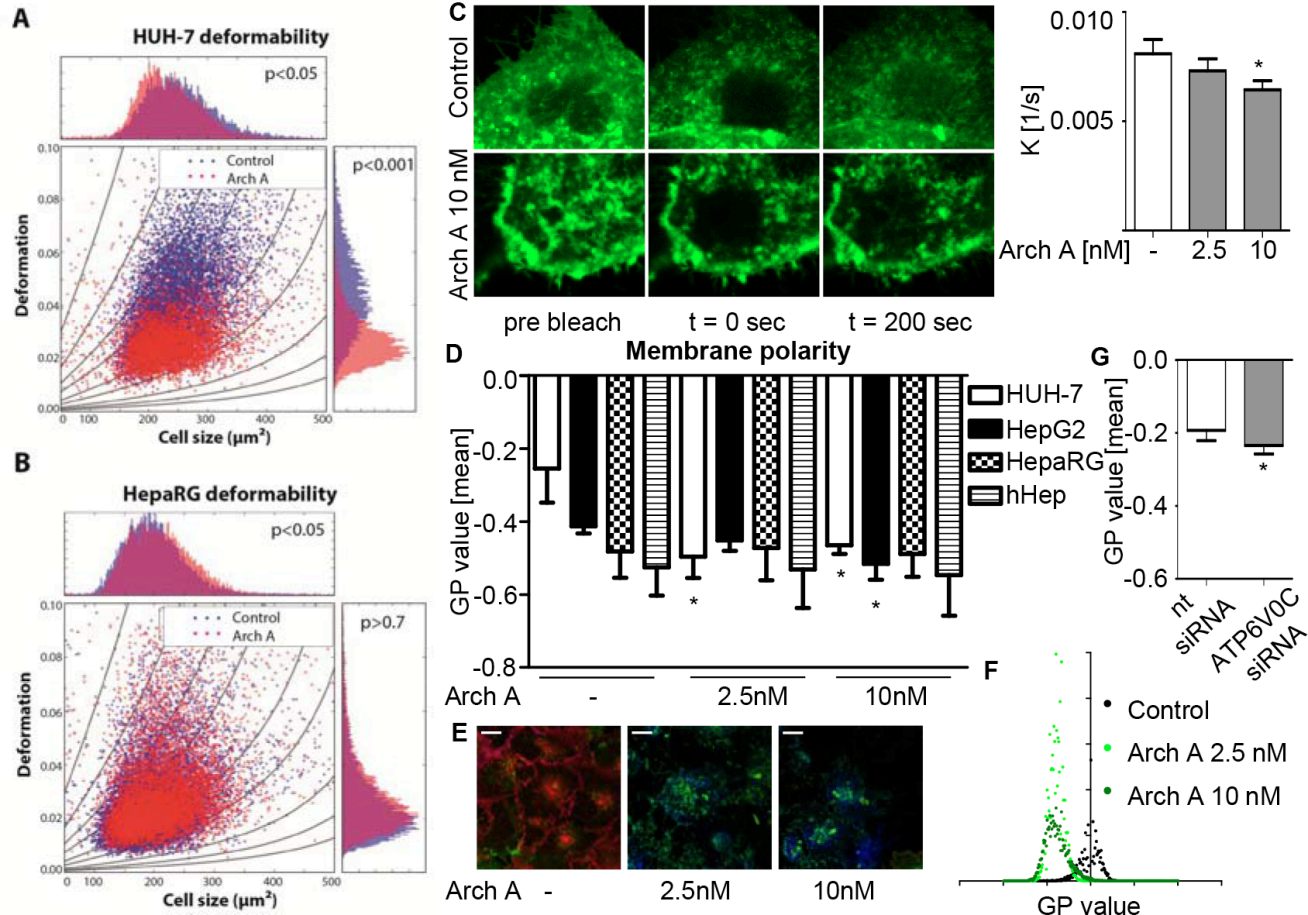
Arch A changes biophysical properties of HCC cells HUH-7 (**A**) and HepaRG (**B**) cells were treated with arch A as indicated (24 h). Deformability was analyzed by RT-DC and is shown for the flow rate of 0.16 μl/s. Graphs represent cell size (cross sectional area) versus deformation (1-circularity) with isoelasticity lines representing areas of identical stiffness. *P*-values of three independent experiments with > 3000 cells per experiment were determined by a likelihood ratio test. (**C**) FRAP of HUH-7 cells transfected with membrane targeted (farnesylated) GFP was analyzed by confocal microscopy. Recovery speed constant K was calculated by non-linear curve fit (Graph Pad Prism). One representative image and diagram of FRAP recovery of three independent experiments are shown. HUH-7, HepG2, HepaRG and hHep were treated with arch A as indicated (24 h) (**D**) and HUH-7 cells were transiently transfected with nt siRNA or siRNA silencing c-subunit of the V-ATPase (72 h) (**G**). Membrane polarity was analyzed by confocal microscopy of live cells stained with di-4-ANEPPDHQ. Representative heat map images (**E**) and histogram (**F**) of GP value distribution of HUH-7 di-4-ANEPPDHQ stainings are shown. Scale bar 20 μM. Bars are the SEM of three independent experiments. *p < 0.05 (One-way ANOVA, Dunnett post test).

The fact that increased cell compliance correlates with cancer cell malignancy [17] and the fact that V-ATPase regulates cholesterol metabolism [13, 14, 21] resulted in the working hypothesis that modulation of V-ATPase by archazolid A might influence cell deformability. Here, we took advantage of a new microfluidic-based technique called real-time deformability cytometry (RT-DC), which allows the measurement of cell deformation while cells pass through a narrow constriction with a rate of 100 cells/sec [22]. RT-DC measurements revealed reduced overall cell deformability upon archazolid A treatment, indicating a stiffening effect of the compound. Blue dots represent control cells, red dots archazolid A treated cells (Figure 1A). Interestingly, this effect seems to be cancer cell specific as the non-malignant hepatocyte cell line HepaRG showed no change in deformation (Figure 1B). As no obvious alterations in the structural organization of the cytoskeleton were observed (Supplementary Figure S1), we further investigated the biophysical properties of the membrane as a possible cause for the altered compliance upon archazolid A treatment.

For this purpose, we measured membrane fluidity in a FRAP assay. We expressed a farnesylated and hence membrane targeted GFP in HUH-7 cells and monitored recovery after bleaching. While untreated cells recovered fast, the repair was much slower in archazolid A treated cells (Figure 1C), indicating a reduced lateral mobility of farnesylated proteins. Furthermore, we investigated membrane polarity by using the membrane-intercalating dye di-4-ANEPPDHQ. This dye undergoes a 60 nm spectral blue shift between disordered and ordered membrane compartments, representing non-raft and cholesterol-rich lipid raft membrane regions, respectively. This allows a quantitative analysis of membrane polarity by generalized polarization (GP) values as described previously [23]. Following archazolid A treatment, GP values, indicating increased membrane polarity, decreased in HUH-7 and HepG2 cells (Figure 1D). This could be visualized by heat map images (Figure 1E) and a shift in the respective GP value distribution histograms (Figure 1F). Knocking down V-ATPase function (siRNA) also led to a GP value reduction (Figure 1G) ensuring a V-ATPase dependent mechanism. Again we observed cancer cell specificity, as GP values of HepaRG and primary human hepatocytes (hHep) (Figure 1D) remained unaltered by archazolid A. These findings clearly reveal that archazolid A specifically alters biophysical characteristics of HCC cells without affecting non-malignant cells.

### V-ATPase inhibition induces lysosomal cholesterol trapping and alterations in cholesteryl-ester profile

**Figure 2:**
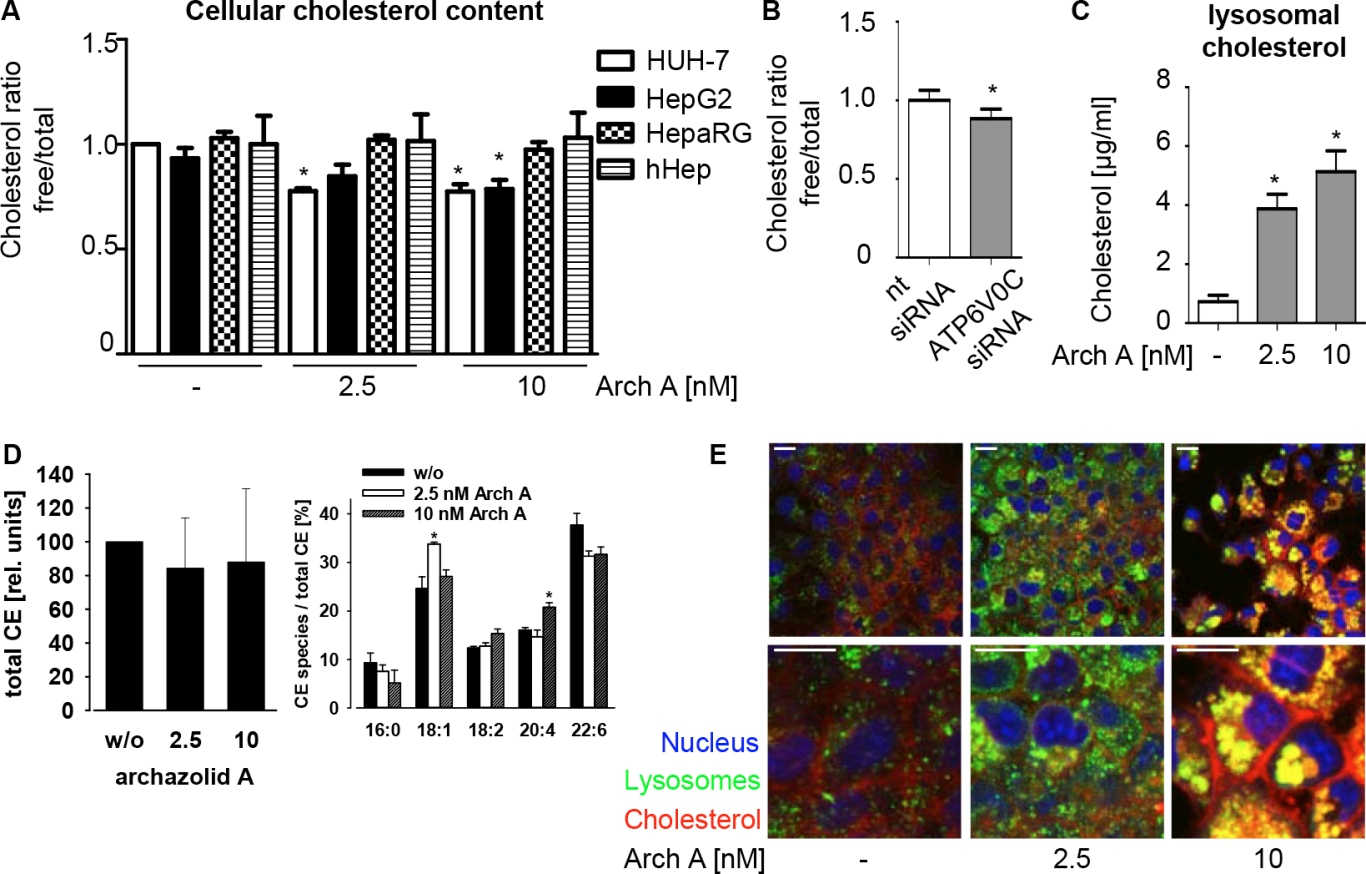
Arch A alters cholesterol metabolism in cancer cells (**A**) Levels of total and free chol of HUH-7, HepG2, HepaRG and hHep cells treated with arch A (48 h) as indicated were assessed using Amplex Red^®^ assay. (**B**) HUH-7 cells were transiently transfected with nt siRNA or siRNA silencing c-subunit of the V-ATPase (72 h) and chol content was analyzed. (**C**) HUH-7 cells were treated with arch A as indicated (48 h) and lysosomes were isolated. Levels of total chol in lysosomes were analyzed. (**D**) HUH-7 cells were treated with arch A (48 h) as indicated, lipids were extracted and cholesteryl ester composition was analyzed by mass spectrometry. (**E**) HUH-7 cells were treated as indicated (24 h), stained for chol (red), lysosomes (green) and nuclei (blue) and analyzed by confocal microscopy. Representative images out of three independent experiments are shown. Scale bar 20 μM. Bars are the SEM of three independent experiments. *p < 0.05 (One-way ANOVA, Dunnett post test).

As the rather unpolar lipid cholesterol is one of the main plasma-membrane components seemingly influenced by the V-ATPase [11], we investigated cellular cholesterol levels upon archazolid A treatment. The enzyme-based Amplex Red^®^ fluorescence assay revealed that the proportion of free cholesterol was significantly diminished upon archazolid A treatment in the HCC cell lines, whereas no changes in cholesterol levels were observed for non-malignant HepaRG cell or hHep (Figure 2A). V-ATPase knock down had similar effects on free cholesterol levels in HUH-7 cells (Figure 2B).

To elucidate the mechanism of archazolid A induced cholesterol depletion, we analyzed lysosomal cholesterol content. The V-ATPase is of crucial importance for lysosomal recycling-function. We previously showed an inhibition of EGF and transferrin receptor recycling by archazolid [13, 14] and could observe a similar effect for the low-density lipoprotein receptor (LDLR) (Supplementary Figure S2). Here, we found that purified lysosomes of treated HUH-7 cells have higher cholesterol levels (Figure 2C), indicating cholesterol trapping. This could also be visualized by a confocal co-staining for cholesterol and the lysosomal marker protein LAMP-1. Control cells displayed a fine dispersion of LAMP-1 and cholesterol within the cell, whereas archazolid A treated cells showed huge accumulations of both stainings (Figure 2E).

Interestingly, ultraperformance liquid chromatography-coupled ESI tandem mass spectrometry (UPLC-MS/MS) revealed alterations in relative composition of cholesteryl-ester (CE) species of archazolid A treated cells, while the total amount of CE remained unchanged (Figure 2D). Together, these data indicate a reduction in free cholesterol levels and a change in the CE profile of the cells due to cholesterol trapping in lysosomes by archazolid A in cancer cells.

### Plasma-membrane cholesterol depletion leads to impaired Ras signaling

**Figure 3:**
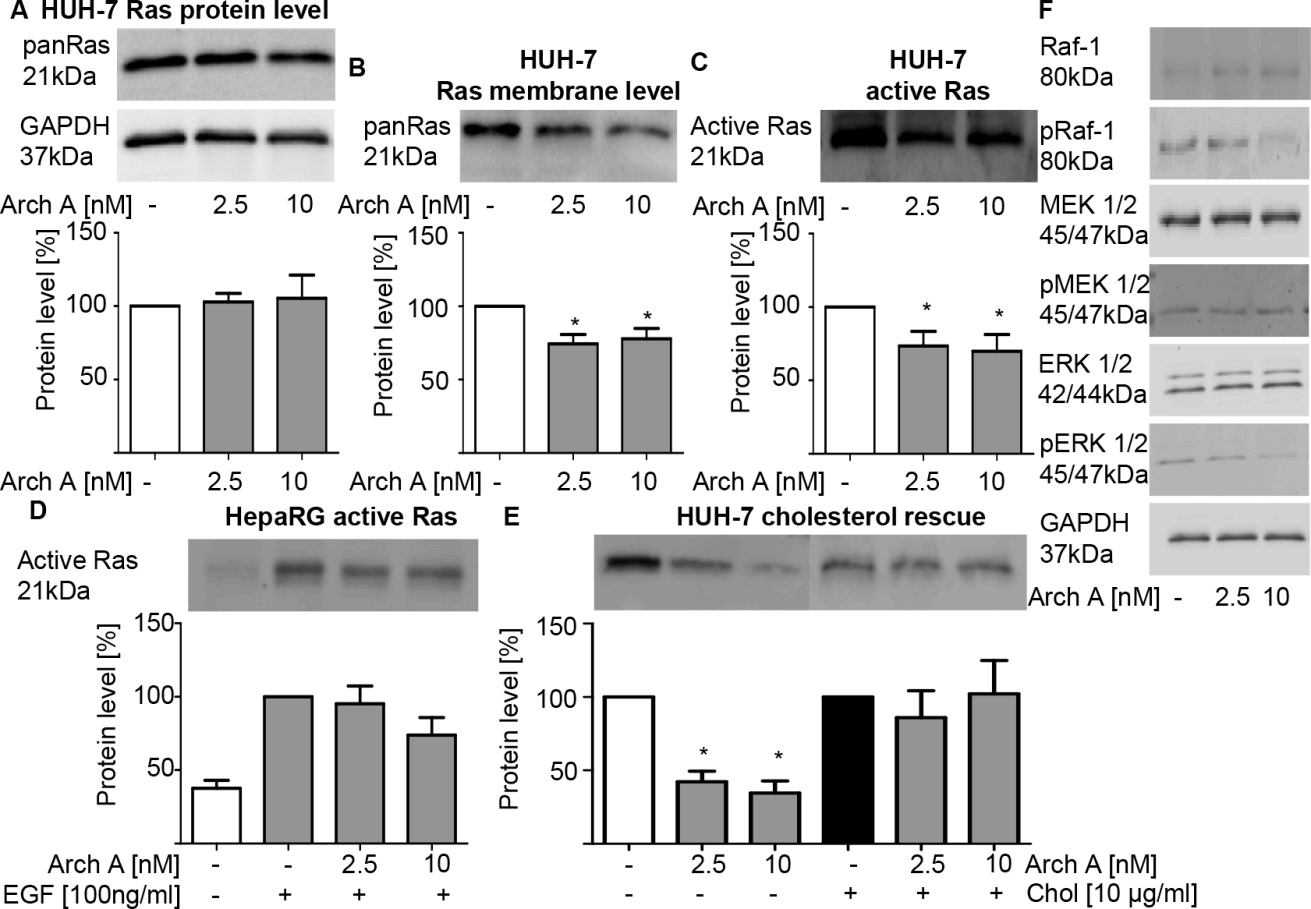
Arch A induced changes lead to reduced Ras/Raf/MEK/ERK signaling (**A**) PanRas protein expression of HUH-7 cells was detected by western blot (WB) upon arch A treatment (48 h). (**B**) PanRas protein levels in membrane fractions of arch A (48 h) treated HUH-7 was detected by WB. (**C, D**) Active Ras was pulled down from cell lysate of arch A treated (48 h) HUH-7 and HepaRG cell lysate and analyzed by WB, respectively. In HepaRG cells Ras signaling was stimulated by EGF (100 ng/ml) treatment 15 min prior to lysis. (**E**) HUH-7 cells were treated with arch A together with or without chol as indicated (48 h). Active Ras was pulled from cell lysates. (**F**) Protein expression of Raf-1, pRaf-1 (Ser338/Tyr341), MEK 1/2, pMEK 1/2 (Ser217/221), ERK 1/2 and pERK 1/2 (Thr202/Tyr204) of HUH-7 cells treated with arch A (48 h) was analyzed by WB. GAPDH served as loading control. Bars are the SEM of quantification of three independent experiments. *p < 0.05 (One-way ANOVA, Dunnett post test).

As cholesterol-rich lipid-rafts are particularly important signaling platforms for the activation of farnesylated proteins, we assessed consequences of archazolid A treatment on the small GTPase Ras. While the overall Ras protein expression of HUH-7 cells remained unaffected (Figure 3A), Ras levels within the plasma membrane significantly decreased (Figure 3B). Additionally, we found significantly less active Ras upon treatment (Figure 3C). The absence of an effect in HepaRG cells (Figure 3D) points to a tumor specific effect.

To confirm membrane cholesterol depletion as cause of decreased Ras activation, we performed a cholesterol rescue experiment. When soluble cholesterol was added to the medium, Ras remained active despite archazolid A treatment (Figure 3E) confirming a cholesterol dependency. Impaired Ras activation is known to have effects on various downstream signaling pathways, especially MEK/Erk and PI3K/Akt. We could show that there is an inhibition of the MEK/ERK pathway shown by decreased phosphorylation of Raf-1, MEK 1/2 and ERK 1/2 in archazolid A treated HUH-7 cells (Figure 3F). Of note, there was no effect on the PI3K/Akt pathway as there were neither changes in the expression of PI3K, Akt or Bad nor in the phosphorylation of Akt and Bad (Supplementary Figure S3).

### Archazolid A strongly inhibits cancer cell proliferation *in vitro* and *in vivo*

**Figure 4:**
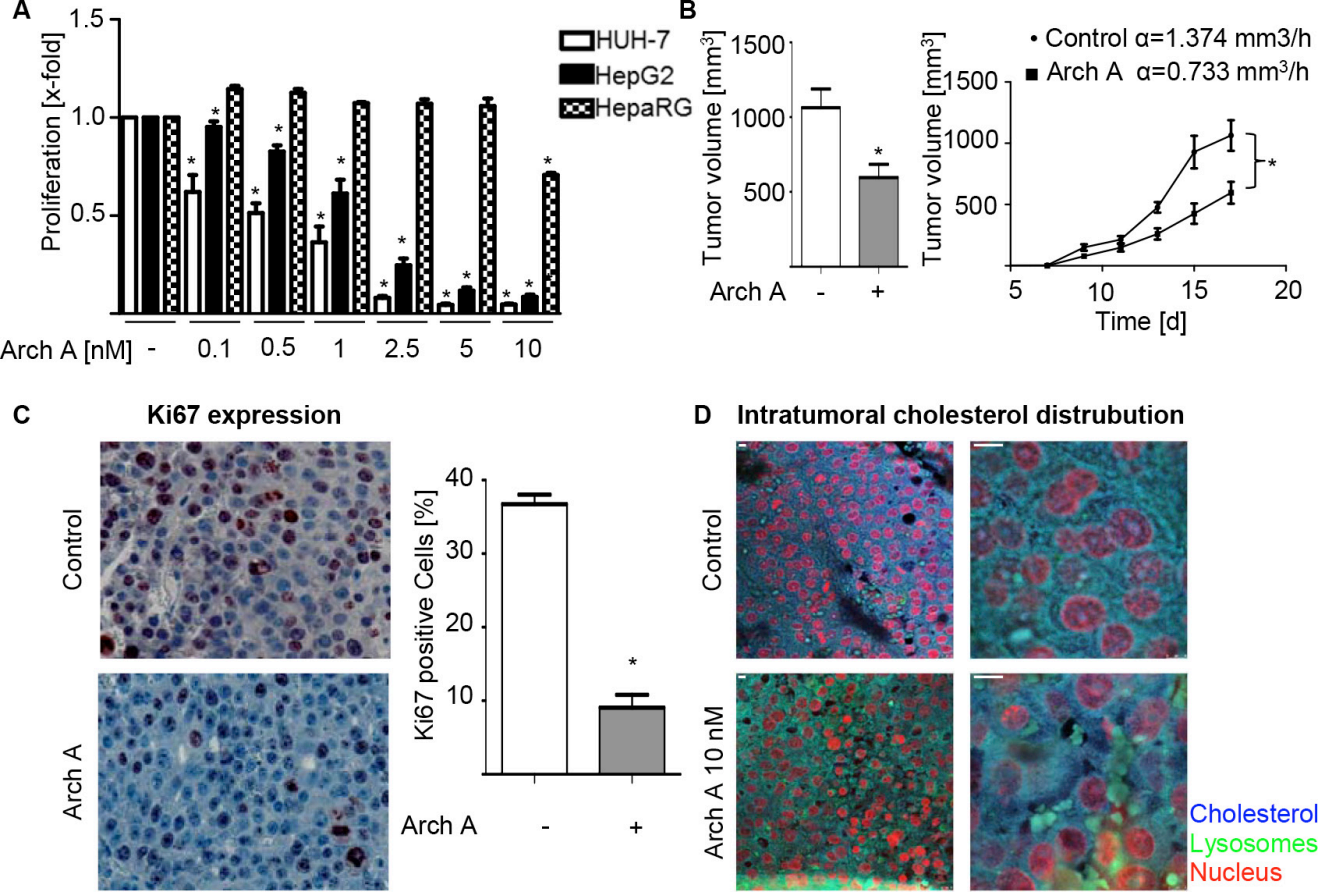
Arch A leads to reduced proliferation *in vitro* and *in vivo* (**A**) Proliferation of HUH-7, HepG2 and HepaRG cells was analyzed by CellTiter Blue assay. (**B**) HUH-7 cells were injected s.c. into the flanks of 16 SCID mice. Mice were divided in two groups and treated daily i.p. with arch or equal amounts of solvent. Tumor volume and growth rates α are indicated (Co α = 1.374 mm^3^/h vs. arch, α 1.30 mm^3^/h). (**C**) Paraffin sections of tumors were stained for Ki67 and nuclei. (**D**) Paraffin sections of tumors were stained for chol (blue) and LAMP-1 (green). Representative images of control and arch-treated mice are shown. Scale bar 20 μM. Bars are the SEM of three independent experiments. *p < 0.05 (unpaired *t*-test).

Due to the fact, that Ras signaling is important for cell proliferation, we investigated archazolid A effects on cell proliferation. Proliferation of HCC cell lines was concentration dependently inhibited by archazolid A in contrast to HepaRG cells (Figure 4A). Importantly, treatment with archazolid A also showed strong effects in an *in vivo* mouse xenograft model. Daily treatment with archazolid A starting at day 7 after tumor-cell injection and lasting until day 17 greatly impaired HCC tumor proliferation, reducing tumor size and growth rate (Figure 4B). Consistent with this finding histological analysis showed that tumors of archazolid A treated mice express less Ki67 (Figure 4C), a marker for proliferation. Additionally, staining of tumor sections for cholesterol and LAMP-1 showed lysosomal cholesterol accumulations as expected by our *in vitro* results (Figure 4D).

Another important feature of invasive cancers is the ability to migrate and invade, for which the cell stiffness is of great importance [24]. Therefore, we analyzed the ability of HUH-7 cells to migrate along a fetal calf serum (FCS) gradient and to invade into Matrigel^®^Matrix in a Boyden Chamber Assay. We found that archazolid A treatment strongly inhibits both, migration (Supplementary Figure S4A) and invasion (Supplementary Figure S4B). Interestingly, this effect could partially be rescued by cholesterol supplementation to the media.

Together, our findings show that archazolid A inhibits the proliferation of HCC cell lines *in vitro* and *in vivo* and influences migration and invasion of cancer cells, mediated by cholesterol restriction to the lysosomes.

## DISCUSSION

In the present study we show, that the V-ATPase inhibitor archazolid A reduces tumor cell proliferation *in vitro* and *in vivo* by modifying the mechanical phenotype of HCC cells. Using an interdisciplinary approach in combining biophysical and cell-biological methods we could reveal a new possible therapeutic strategy for specifically targeting HCC, while leaving non-malignant cells unaffected.

Until recently, the main aim in targeting cancer was to directly alter signaling pathways that are responsible for proliferation, invasion and metastasis. However, it becomes more and more clear, that these processes greatly depend on the biomechanical and biophysical aspects of the cells and their environment [25]. Hence, recent research focused on the role of cancer cell mechanics. Several studies show that cancer cells display an altered mechanical phenotype, compared to their non-malignant counterparts. Lin et al. found that different cancer cell lines of breast, bladder, cervical and pancreatic cancer are each softer than their respective, non-malignant counter-parts. They also showed that malignant cells lose their ability to sense and adapt to stiffness changes of the extracellular environment, possibly enabling them to increased migration and invasion. [17] In line with these findings, the softness of tumor cell lines and patient cancer cells correlates with invasiveness [26]. Accordingly, we could reveal that the HCC cell line HUH-7 is more deformable than the non-malignant hepatocyte cell line HepaRG, reinforcing increased compliance as a characteristic of cancer cells in general. These data are supported by the finding of others, which show changes in stiffness for HCC compared to non-malignant tissue [27]. Treatment of HCC cell lines with the V-ATPase inhibitor archazolid A increased cell stiffness compared to control HCC cells and importantly, leaves non-malignant cells unaffected. This displays a new option in treating HCC by specifically addressing biomechanical properties of liver cancer cells.

Cell stiffness is determined by the components of the cytoskeleton, that have been extensively studied, and by the composition of membranes, of which less is known [28–30]. This is due to the fact that membranes are composed of thousands of different lipid species, which only recently moved into the center of interest due to advances in chromatographic and mass spectrometric lipid analytics as well as imaging techniques, though modifying options are still largely missing [31]. Nevertheless, evidence shows that lipid metabolism is frequently aberrant and important for cancer cells, especially in terms of signaling and cytoskeletal adhesion [32–34]. Cholesterol is an essential cellular lipid and as such, is necessary for the regulation of membrane fluidity, vesicle trafficking, endocytosis and receptor signaling. In the context of HCC, it has been reported that cholesterol metabolism is aberrant and seems to be play a major role in the malignant phenotype [35–37]. For instance, elevations in overall or mitochondrial cholesterol content in primary tumor cells or HCC cell lines were linked with chemotherapy resistance and protection from apoptosis [37]. It has been reported that cholesterol depletion can increase cell stiffness and regulate membrane fluidity [38]. However, to our knowledge nothing is known on how cellular biophysical properties based on lipid alterations influence proliferation of human cells. Interestingly, Atilla-Gokcumen et al. recently found first evidence, that cells tightly regulate lipid species and localization during the cell cycle by excessive feedback loops, leading to variations in cell stiffness along the cell cycle [39].

Here we show that inhibiting V-ATPase function not only traps cholesterol in lysosomes but also decreases the levels of free cholesterol and depletes cholesterol from the plasma-membrane. The V-ATPase is already known to play an important role in cancer cell apoptosis, metastasis, receptor recycling and metabolism [12–14]. Interestingly, it has also been implicated in cholesterol metabolism recently. Hamm et al. proposed that interference of archazolid with cholesterol metabolism is a main resistance mechanism of bladder cancer cells to the drug [21]. According to Hamm et al.'s data we demonstrate an upregulation of SREBP-2, HMGCR and LDLR gene transcription (Supplementary Figure S5), however, we draw a different conclusion. We propose that increased transcription of cholesterol regulating genes is a feedback regulation owing to cholesterol trapping caused by archazolid and show that archazolid leads to cholesterol-depletion of the plasma-membrane which alters important biophysical properties of cancer cells – stiffness and polarity of the plasma membrane.

Cholesterol enriched membrane compartments act as the major signaling platforms for a variety of signaling pathways, and a balanced lipid composition is inevitable for the constant activation of many pro-survival signaling mechanisms in tumors. The small GTPase Ras is a member of an important family of membrane-targeted signaling molecules, which is a well-known oncogene mutated in 20% of all tumors [20]. In HCC, excessive Ras activation has been reported [40], which may result from aberrant upstream signaling or inactivation of tumor suppressor genes [41]. Aberrant Ras signaling in tumors displays a poor prognosis factor for cancer patients. The lipid composition of the plasma membrane is crucial for the activation of Ras, as it is modified by post-translational farnesylation and palmitoylation, targeting Ras to specific cholesterol-rich membrane locations [20, 42, 43]. We could show that upon V-ATPase inhibition the activation of Ras is diminished, leading to impaired downstream signaling namely Raf/MEK/ERK and reduced proliferation *in vitro* and *in vivo*. This could be shown to result from cholesterol depletion as addition of free cholesterol could restore Ras activation.

**Figure 5:**
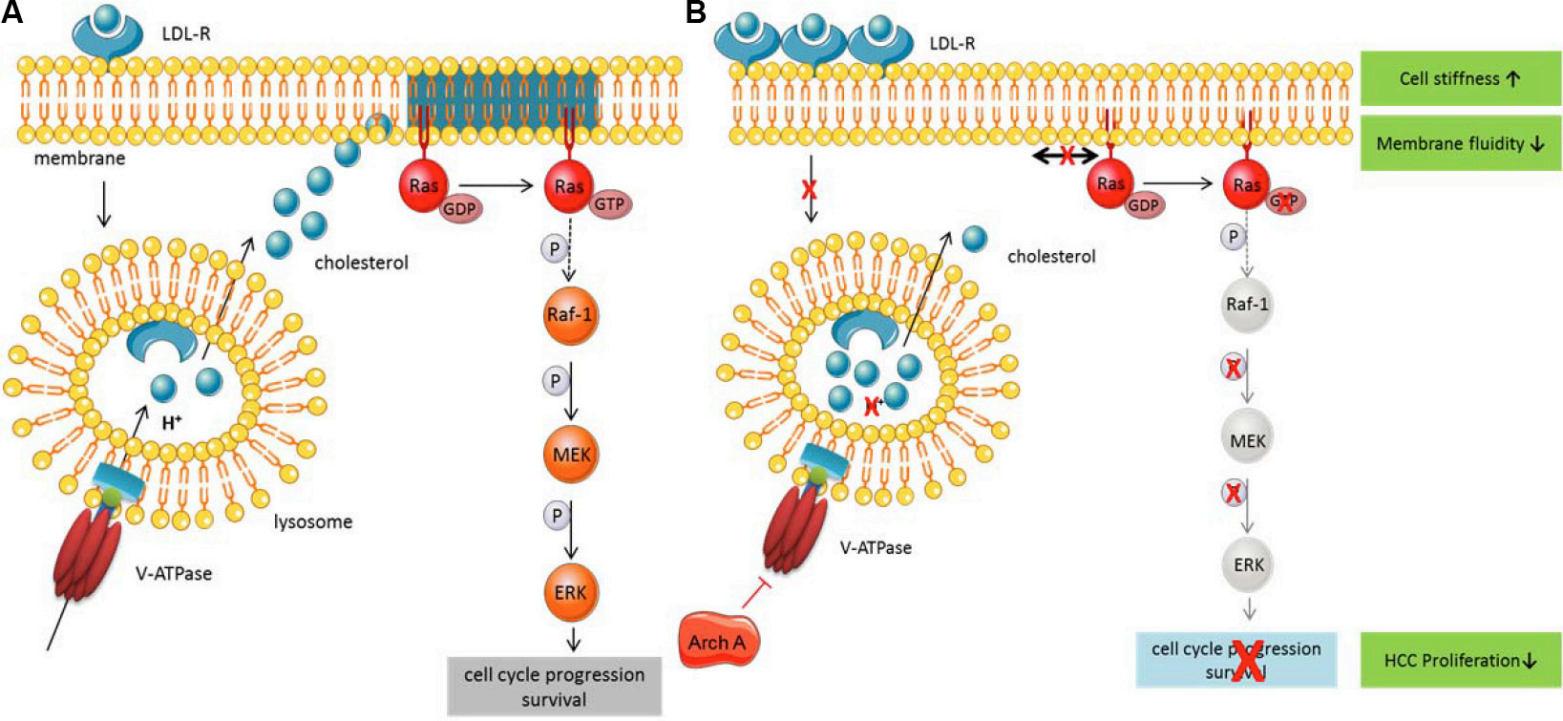
Archazolid, a novel, bidirectional approach in targeting HCC (**A**) Under physiological conditions low-density lipoprotein (LDL) binds to its receptor (LDL-R) and is internalized. The V-ATPase acidifies the endo-lysosome, leading to LDL dissociates from the receptor and cleavage. Free cholesterol is then released into the cytosol and is used as building block and for the integration into membranes. Ras is a membrane-bound small GTPase mainly localized in cholesterol-enriched membrane microdomains, where it can be activated. Ras in turn activates different signaling pathways leading to proliferation and survival. (**B**) Upon inhibition of the V-ATPase by Archazolid, acidification of the endo-lysosome is inhibited and cholesterol accumulates within the lysosomes. The lack of free cholesterol leads to cholesterol depletion of the membrane and subsequently a disruption of cholesterol-enriched microdomains and a change in membrane properties. As a counsequence, cholesterol microdomain-dependent Ras cannot be activated anymore and downstream signaling is inhibited, leading to reduced proliferation.

In our opinion, archazolid displays a novel, bidirectional approach in targeting HCC. The specificity of the effects on cholesterol metabolism and signaling for HCC cells renders V-ATPase inhibition as an interesting apporach in cancer therapy. We provide evidence that by V-ATPase inhibition cholesterol is depleted from the plasma membrane. On the one hand, the concomitant changes in the membrane composition apparently increase cell stiffness and reduced membrane fluidity – two major biophysical characteristics of membranes. These changes most probably account for reduced migration and invasion. On the other hand, proliferative signaling is impaired by diminished Ras activation (Figure 5). As a consequence tumor cell proliferation is greatly reduced *in vitro* and *in vivo*. Especially the fact that archazolid is able to restrict cholesterol also in a mouse tumor xenograft model suggests a potential for clinical application. In conclusion, in this study we show that the anti-cancer agent archazolid changes HCC cell physical properties through lysosomal cholesterol trapping caused by V-ATPase inhibition. This leads to an inhibition in mitogenic signaling and subsequently diminished proliferation of HCC cells, while non-malignant hepatocytes remain unaffected, which we conclude is a crucial part of the anti-tumoral activity of V-ATPase inhibitors.

## MATERIALS AND METHODS

### Compounds and cell culture

HUH7 and HepG2 cells were obtained from Japanese Collection of Research Bioresources (JCRB) and German Research Centre of Biological Material (DSMZ) (ACC180), respectively. Cell line STR profiling was performed. HCC cells were grown in DMEM (PAN-Biotech GmbH, Aidenbach, Germany) supplemented with 10% fetal calf serum (FCS). HepaRG^TM^ cells were obtained from Life Technologies. Cells were plated and maintained in Williams’ medium E supplemented with GlutaMAX^TM^ and HepaRG™ Thaw, Plate, & General Purpose Medium Supplement (Thaw, Plate, & General Purpose Working Medium) purchased from Life Technologies. For metabolism studies, cells were seeded in Thaw, Plate, & General Purpose Working Medium, which was replaced by in Williams’ medium E supplemented with GlutaMAX^TM^ and HepaRG™ Maintenance/Metabolism Medium Supplement (Metabolism Medium) after 24 h. Thereafter medium was renewed every 3 days. Experiments were performed after 7 days of cell maintenance in Metabolism Medium. Hepatocyte tissue samples (hHep) and annotated data were obtained and experimental procedures were performed within the framework of the non-profit foundation HTCR, including the informed patient's consent. All cells were cultured under constant humidity at 37°C and with 5% CO_2_ in an incubator. All culture flasks, multiwell-plates and dishes were first coated with collagen G (0.001% in PBS) before seeding the cells. Archazolid A was provided by Rolf Müller (Saarland University) and was dissolved in dimethylsulfoxide (DMSO) (Sigma-Aldrich). All cell lines were frequently tested for mycoplasma contamination.

### Transient transfection with small interfering RNA (siRNA)

For silencing experiments, cells were seeded 24 h prior to transfection with siRNA using DharmaFECT^TM^ transfection reagents and manufacturer's protocol (Dharmacon^TM^, GE Healthcare). ATP6V0C was silenced using ON-TARGETPlus SMARTpool siRNA (2 mg) from Dharmacon^TM^ and non-targeting siRNA (nt siRNA) as a control.

### Proliferation

Proliferation was assessed with the CellTiter-Blue^®^ Cell Viability Assay (Promega, Madison, WI, USA). Therefore 5,000 cells/well were seeded into 96-well plates and allowed to adhere overnight. Before stimulation initial metabolic activity was determined and cells were treated as indicated for 72 h. 1 h before end of stimulation time CellTiter-Blue^®^ Reagent was added and the absorbance at 590 nm was measured in a Sunrise ELISA reader (Tecan, Maennerdorf, Austria) and is proportional to the cell number.

### Confocal microscopy

To stain HUH7 cells for confocal microscopy 30,000 cells/well were seeded on IBIDI μ-slides (IBIDI, Martinsried, Germany) one day before treatment with archazolid (2.5/10 nM, 24 h). After treatment, cells were washed with PBS, fixed with 3% Paraformaldehyde (PFA) for 30 min, permeabilized with 0.1% Triton-X and unspecific binding was blocked with 2% BSA. Subsequently lysosomal marker protein LAMP-1 was stained with specific antibodies (Developmental Studies Hybridoma Bank) for 2 h at 25°C and secondary antibody (AlexaFluor^®^488, MolecularProbes) for 45 min at 25°C. Cholesterol was stained with 50 μg/ml filipin (Sigma Aldrich) for 2 h at 25°C, together with TO-PRO^®^3 (Life Technologies) staining of nuclei (Supplementary Table S1and S2). Cells were washed and mounted with FluorSaveTM Reagent mounting medium (Beckman Coulter) and covered with a glass coverslip. Images were taken by confocal microscopy (Leica TCS SP 8 SMD, Wetzla, Germany).

### Analysis of membrane polarity

For analysis of membrane polarity, 20,000 cells/well were seeded on IBIDI μ-slides (IBIDI) 24 h prior to stimulation. The cells were treated as indicated for 24 h. Subsequently 10 μM of the dye di-4-ANEPPDHQ in DMEM without FCS were added for 30 min at 37°C. Live cell imaging was performed as described previously [44] using a Leica TCS SP 8 SMD confocal microscope with a top stage incubator (Oko Lab, Ottaviano, Italy). For analysis of the images a macro for ImageJ (ImageJ 1.46r, NIH, USA) based on the one provided by Owen et al. [44] was used.

### Fluorescence recovery after photo bleaching

24 h prior to treatment, HUH7 cells were transfected with a plasmid coding for farnesylasted GFP (pAcGFP-F, Clontech, CA, USA) using Amaxa^®^ Cell Line Nucleofector^®^ Kit T (Lonza Cologne AG, Cologne, Germany) employing program T 28 and subsequently seeded onto IBIDI μ-slides (IBIDI). Cells were treated with archazolid (2.5/10 nM, 24 h) and FRAP assay was performed using a Leica TCS SP 8 SMD confocal microscope with a top stage incubator (Oko Lab, Ottaviano, Italy). A defined region of interest was bleached with high laser power and recovery of the GFP signal was monitored by recording 60 post bleach images every 10 s.

### Cholesterol measurement

Cellular cholesterol levels were measured using the Amplex^®^ Red Cholesterol Assay Kit (Molecular Probes) according to manufacturer's protocol. Therefore cells were treated as indicated for 48 h, detached and either homogenized right away in a lipid extraction solution containing chloroform, isopropanol and IGEPAL CA-630 (7:11:0.1, Sigma) via sonication, or homogenization was performed on lysosomes isolated as described previously [45]. After centrifugation (13,000 × g, 10 min) organic phase was air dried at 50°C for 10 min to remove chloroform. Remaining organic solvent was removed by vacuum at 30°C over 30 min. Dried lipids were dissolved in 1× assay reaction buffer and mixed 1:1 with a working solution containing 300 μM Amplex^®^ Red reagent, 2 U/ml horseradish peroxidase (HRP), 2 U/ml cholesterol oxidase and in case of total cholesterol measurement 0.2 U/ml cholesterol esterase. After incubation for 30 min at 37°C fluorescence was measured using a Sunrise ELISA reader (Tecan).

### Preparation of cell lysates

For preparation of whole cell lysate, cells were collected by centrifugation, washed with ice-cold PBS, and lysed for 30 min in 1% Triton X-100, 137 mM NaCl, and 20 mM Tris-Base (pH 7.5) with the protease inhibitor complete (Roche). Lysates were centrifuged at 10,000 × g for 10 min at 4°C. For preparation of membrane fractions, cells were washed with ice-cold PBS, buffer A (250 mM Sucrose, 20 mM HEPES,10 mM KCl, 1.5 mM MgCl_2_, 1 mM EDTA, 1 mM EGTA, 1 mM DTT, protease inhibitor complete) was added, cells were scraped off and passed through a 25 Ga needle. Cell lysate was incubated on ice for 20 min and centrifuged at 14000 × g, 4°C for 20 min. The supernatant was collected and centrifuged at 100,000 × g, 4°C for 1 hour. The supernatant was collected as cytosolic fraction and the pellet was dissolved in buffer B (buffer A supplemented with 10% glycerol and 0.1% SDS), representing the membrane fraction. All fractionation steps were performed at 4°C.

### Western blot

Equal amounts of protein were separated by SDS-PAGE and transferred nitrocellulose membranes (Hybond-ECL^TM^, Amersham Bioscience). Membranes were blocked with 5% fat-free milk powder in PBS containing 0.1% Tween 20 for 2 h and incubated with specific antibodies against ERK 1/2 (Cell signalling), pERK 1/2 Thr202/Tyr204 (Cell signaling), GAPDH (Santa Cruz), MEK 1/2 (Santa Cruz), pMEK 1/2 Ser217/221 (Cell signaling), panRas (Santa Cruz), Raf 1 (Santa Cruz) and pRaf-1 Ser 338/Tyr 341 (Santa Cruz) over night at 4°C. Proteins were visualized by secondary antibodies conjugated to horseradish peroxidase (HRP) and freshly prepared ECL solution, containing 2.5 mM luminol (Supplementary Tables S3 and S4). Chemiluminescence signal was detected with the ChemiDoc^TM^ Touch Imaging System (Bio-Rad, Munich, Germany).

### Ras activity assay

Ras activation status of the cells was determined using the Ras Assay Kit (ab128504, Abcam), according to manufacturer's protocol. Cells were seeded 24 h prior to treatment with archazolid 2.5/10 nM, 48 h) and cholesterol (10 μg/ml, 48 h). After stimulation medium was aspirated off, ice-cold lysis solution, containing GST-Raf-RBD which specifically binds to active GTP-bound Ras, was added and cells were scraped off, using a rubber police man. After centrifugation (12,000 × g, 4°C), supernatant was mixed with Glutathione-Sepharose-Slurry beads, that bind to GST-Raf-RBD and incubated under constant mixing for 30 min at 4°C. After incubation, beads were spinned down and drained well, mixed with SDS-containing sample buffer for SDS-PAGE, denatured for 10 min at 95°C and subjected to Western Blotting as described above. Protein loading on the gel was determined using 0.5% trichloroethanol (Sigma) polyacrylamide gels as described before [46]. Primary antibody detecting panRas was provided in the kit and secondary antibody goat-anti-mouse IgG, conjugated to HRP were used (Santa Cruz).

### Cholesteryl ester analysis

HUH-7 cells were treated with archazolid A (2.5/10 nM, 48 h) and collected by centrifugation. Cell pellet was frozen in liquid nitrogen and stored at –80°C until use. The pellet was resuspended in MeOH, chloroform was added and finally PBS. Cells were then centrifuged at 4000 rpm for 5 min and lower chloroform phase was collected. The chloroform was evaporated for 20 min at 30°C and dried lipids were dissolved in MeOH. After centrifugation at 1500 rpm for 5 min, supernatant was diluted with MeOH, centrifuged again at 1500 rpm for 5 min and analysed by LC-MS/MS, as described previously [47].

### Real-time deformability cytometry

For RT-DC measurements the experimental setup has been described earlier [22]. Cells were trypsinzed and resuspended to a final concentration of about 3 × 10^6^ cells/ml in 0.5% methylcellulose solved in PBS. To achieve cell deformation the cell suspension was pumped through a microfluidic chip containing a constricted channel of 30 μm × 30 μm at flow rates of 0.16 μl/s, 0.24 μl/s and 0.32 μl/s. As a reference, non-deformed cells were measured outside the channel in the reservoir where cell deformation does not take place. Cell size (cross-sectional area) and deformation (1 – circularity) was determined in real-time for > 3000 cells per experiment at rates of 100 cells/sec. Isoelasticity lines were assessed as reported elsewhere [48]. Statistical analysis was performed by applying linear mixed effects models. Therefore, a fixed effects model is extended by a random effect term that can be used to account for error induced by the experimental design. The archazolid A treatment was considered as a binary fixed effect whereas biological variations between experiments were taken as a random effect. We allowed the model to fit random intercepts to attribute for variations in the mean values of the control group as well as random interslopes to account for variable differences between the control and the archazolid A-treated group. *P*-values were calculated by a likelihood ratio test.

### *In vivo* HUH-7 xenograft mouse model

Sixteen female SCID mice (Charles River „CB17/lcr-PrkdcSCID/lcrlcocrl”) were locally shaved and 3 × 10^6^ HUH-7 cells were injected subcutaneously into the flank of each mouse. Mice were divided into two groups and treated intraperitoneally with 0.2 mg/kg archazolid in 5% DMSO/10% solutol/PBS or equal amounts of 5% DMSO/10% solutol/PBS. Mice were treated daily. Measurement of tumors was done every 2 to 3 days with a caliper, using the formula a × b^2^/2. The average tumor volumes of the two groups were compared over time. IHC analysis of tumor tissue sections was performed as described previously [49] using anti-LAMP1-antibody (Abcam), filipin (Sigma Aldrich), anti-Ki67-antibody and haematoxylin (Sigma Aldrich). Animal experiments were approved by the District Government of Upper Bavaria in accordance with the German animal welfare and institutional guidelines.

## SUPPLEMENTARY MATERIALS TABLES AND FIGURES



## Abbreviations

arch: archazolid
CE: cholesteryl ester
FRAP: fluorescence after photo-bleaching
GFP: green fluorescent protein
GP: generalized polarization
hHep: primary human hepatocytes
HCC: hepatocellular carcinoma
LDL(R): low-density lipoprotein (receptor)
RT: DC-real-time deformability cytometry.
