# Supplementary material for: V-ATPase inhibition increases cancer cell stiffness and blocks membrane related Ras signaling - a new option for HCC therapy
Source: Oncotarget. 2016 Dec 28;8(6):9476–87. doi: 10.18632/oncotarget.14339 (PMC5354746; doi:10.18632/oncotarget.14339)
Supplement: Supplementary file 1 [file oncotarget-08-9476-s001.pdf]

V-ATPase inhibition increases cancer cell stiffness and blocks membrane related Ras signaling - a new option for HCC therapy

Supplementary Materials

MATERIALS AND METHODS

Confocal microscopy – actin staining

To stain HUH7 cells for confocal microscopy 30,000 cells/well were seeded on IBIDI µ-slides (IBIDI, Martinsried, Germany) one day before treatment with archazolid (2.5/10 nM, 24 h). After treatment, cells were washed with PBS, fixed with 3% Paraformaldehyde (PFA) for 30 min, permeabilized with 0.1% Triton-X and unspecific binding was blocked with 2% BSA. Subsequently, actin was stained with rhodamine-phalloidin (Life Technologies) and nuclei with Hoechst 33342 Bisbenzimide (Sigma Aldrich) for 45 min at 25°C. Cells were washed and mounted with FluorSaveTM Reagent mounting medium (Beckman Coulter) and covered with a glass coverslip. Images were taken by confocal microscopy (Leica TCS SP 8 SMD, Wetzla, Germany).

Confocal microscopy – LDL-R and LDL internalization

30,000 cells/well were seeded, treated with archazolid A (2.5/10 nM, 24 h) and starved for 1.5 h or 2 h prior to LDL-R or LDL internalisation assay, respectively. For LDL internalisation assay, 10 µg/ml DilLDL (Molecular Probes) in DMEM were added for 10 min at 37°C. Cells were subsequently washed, fixed with 4% paraformaldehyde for 20 min, washed again and mounted with FluorSaveTM Reagent mounting medium (Beckman Coulter) and covered with a glass coverslip.

In case of LDL-R internalisation, anti-LDL-R antibody (Santa Cruz) in DMEM containing 0.1%BSA was added for 45 min at 4°C. Subsequently cells were washed, incubated for 1 h at 37°C and 1 µM Phorbol-12-Myristate-13-Acetate (PMA) was added for 30 min at 37°C. Cells were then washed, fixed with 4% PFA for 15 min, permeabilized with 0.1% Triton-X and stained with fluorescent secondary antibody (AlexaFluor®488, MolecularProbes) for 1 h at 25°C. After washing, cells were mounted with FluorSaveTM Reagent mounting medium (Beckman Coulter) and covered with a glass coverslip. Images were taken by confocal microscopy (Leica TCS SP 8 SMD, Leica, Mannheim, Germany).

Boyden chamber assay

1×10^5 treated HUH-7 cells were placed on top of the Transwell® chamber (Corning) in media without FCS. The Transwell® chamber was either coated with collagen G (0.001% in PBS) for migration experiments or filled with Matrigel®Matrix (Corning) 1:10 in media for invasion experiments. A 24-well plate was filled with medium with 10% FCS. Transwell® chambers were placed in it and incubated for 16 h in case of migration and 48 h in case of invasion. For negative control (- control) media without FCS was filled in the 24-well plate. Migrated or invaded cells were fixed and stained with crystal violet/methanol. The top of the Transwell® chamber was cleaned and pictures were taken. Migrated and invaded cells were quantified with Image J (National Institutes of Health).

Supplementary Table S1: Primary antibodies for confocal microscopy

Antigen	Company	Cat. No.
LAMP-1	Developmental Studies Hybridoma Bank	H4A3
LDL-R	Santa Cruz	sc-18823

Supplementary Table S2: Secondary antibodies and dyes for confocal microscopy

Antigen	Company	Cat. No.
Mouse IgG (AlexaFluor 488 conjugate)	Life Technologies	A - 11001
TO-PRO®3	Life Technologies	T3605
Filipin	Sigma Aldrich	F4767
di-4-ANEPPDHQ	Life Technologies	D36802
Rhodamine-Phalloidin	Life Technologies	R-415
DilLDL	Life Technologies	L3482
Hoechst 33342	Sigma Aldrich	H6024

Supplementary Table S3: Primary antibodies for western blotting

Antigen	Company	Cat. No.
ERK 1/2	Cell signaling	9102
pERK 1/2 Thr202/Tyr204	Cell signaling	9106
GAPDH	Santa Cruz	sc-69778
MEK 1/2	Santa Cruz	sc-436
pMEK 1/2 Ser217/221	Cell signaling	9121
panRas	Santa Cruz	sc-14022
Raf 1	Santa Cruz	sc-7267
pRaf-1 Ser 338 / Tyr 341	Santa Cruz	sc-28005-R
PI3K	Upstate	06-195
Akt	Cell Signaling	9272
pAkt (Ser473)	Santa Cruz	sc-7985-R
Bad	Cell signaling	9292
pBad (Ser136)	Cell siganling	9295

Supplementary Table S4: Secondary antibodies for western blotting

Antigen	Company	Cat. No.
Mouse IgG (HRP conjugate)	Santa Cruz	sc-2005
Rabbit IgG (HRP conjugate)	Bio-Rad	172-1019

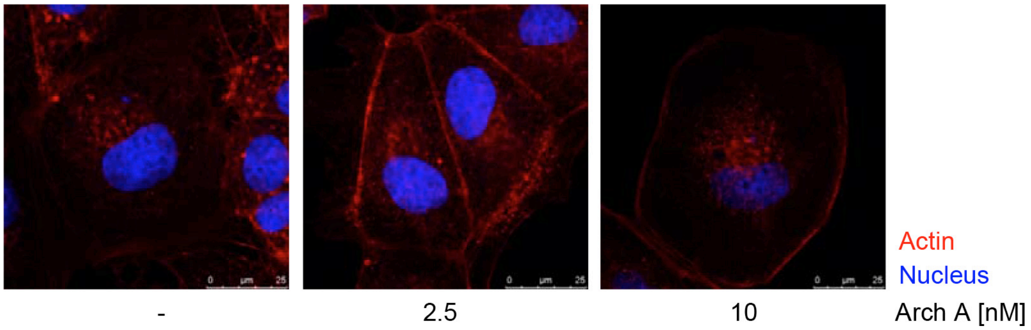

Supplementary Figure S1: Arch A shows no influence on actin cytoskeleton. HUH-7 cells were treated with arch A as indicated (24 h) stained for actin (red) and nuclei (blue) and analyzed by confocal microscopy. Representative images out of three independent experiments are shown.

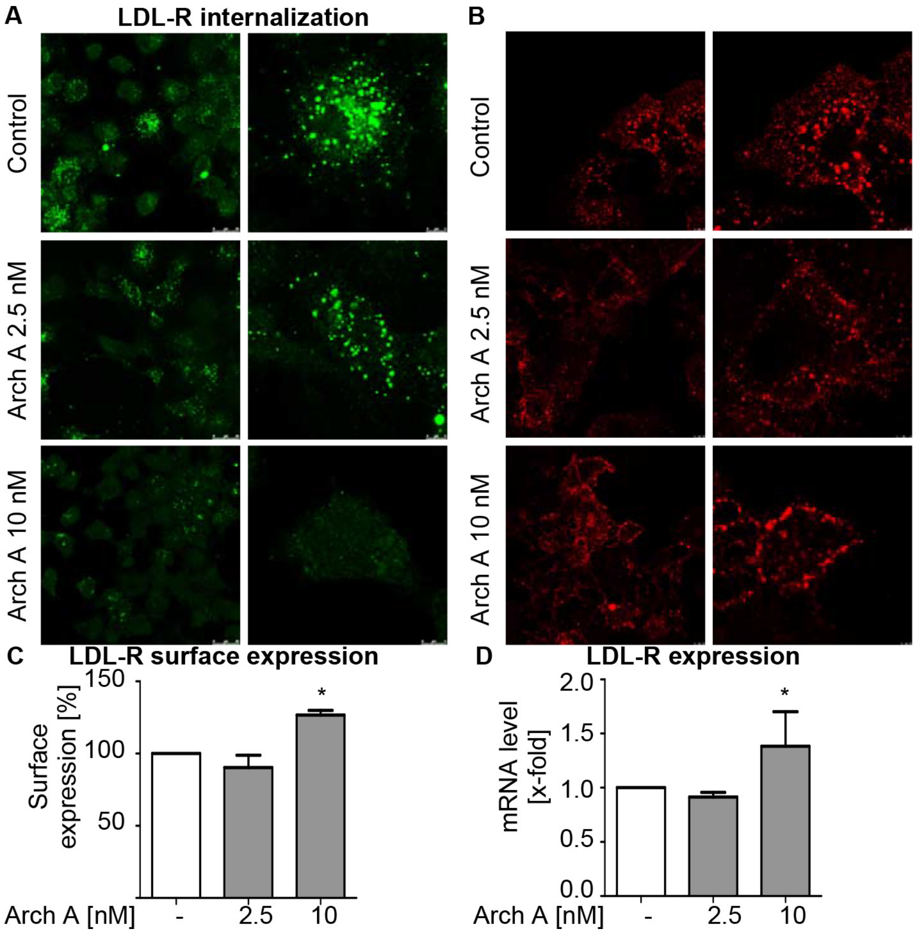

Supplementary Figure S2: Arch A interferes with LDL-R internalization. (**A**) HUH-7 cells were treated with arch A (24 h) and subsequently starved for 2 h. After incubation with PMA (1 h) internalized low-density lipoprotein receptor (LDL-R) was visualized by antibody staining and analysed by confocal microscopy. (**B**) HUH-7 cells were treated with arch A (24 h) and subsequently starved for 1.5 h. Internalization of DilLDL (fluorescently labeled soluble LDL) was analysed by confocal microscopy. (**C**) LDL-R surface expression of HUH-7 cells was visualized by antibody staining and analysed by flow cytometry after arch A treatment (24 h). (**D**) Relative mRNA expression levels of LDL-R in HUH-7 cells were detected with the AB 7300 RealTime PCR system. Bars are the SEM of three independent experiments. *$p < 0.05$ (Repeated measures ANOVA, Dunnett post test) Representative images out of three independent experiments are shown.

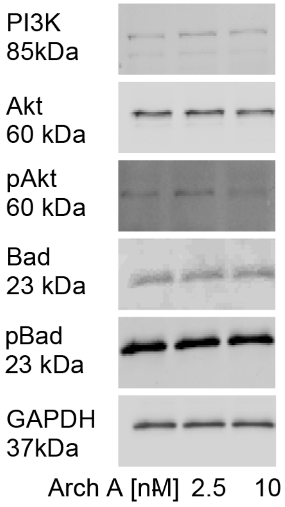

Supplementary Figure S3: Arch A does not affect PI3K/Akt pathway Protein expression of PI3K, Akt, pAkt (Ser473), Bad and pBad (Ser136) of HUH-7 cells treated with arch A (48 h) was analyzed by WB. GAPDH served as loading control. Representative images out of three independent experiments are shown.

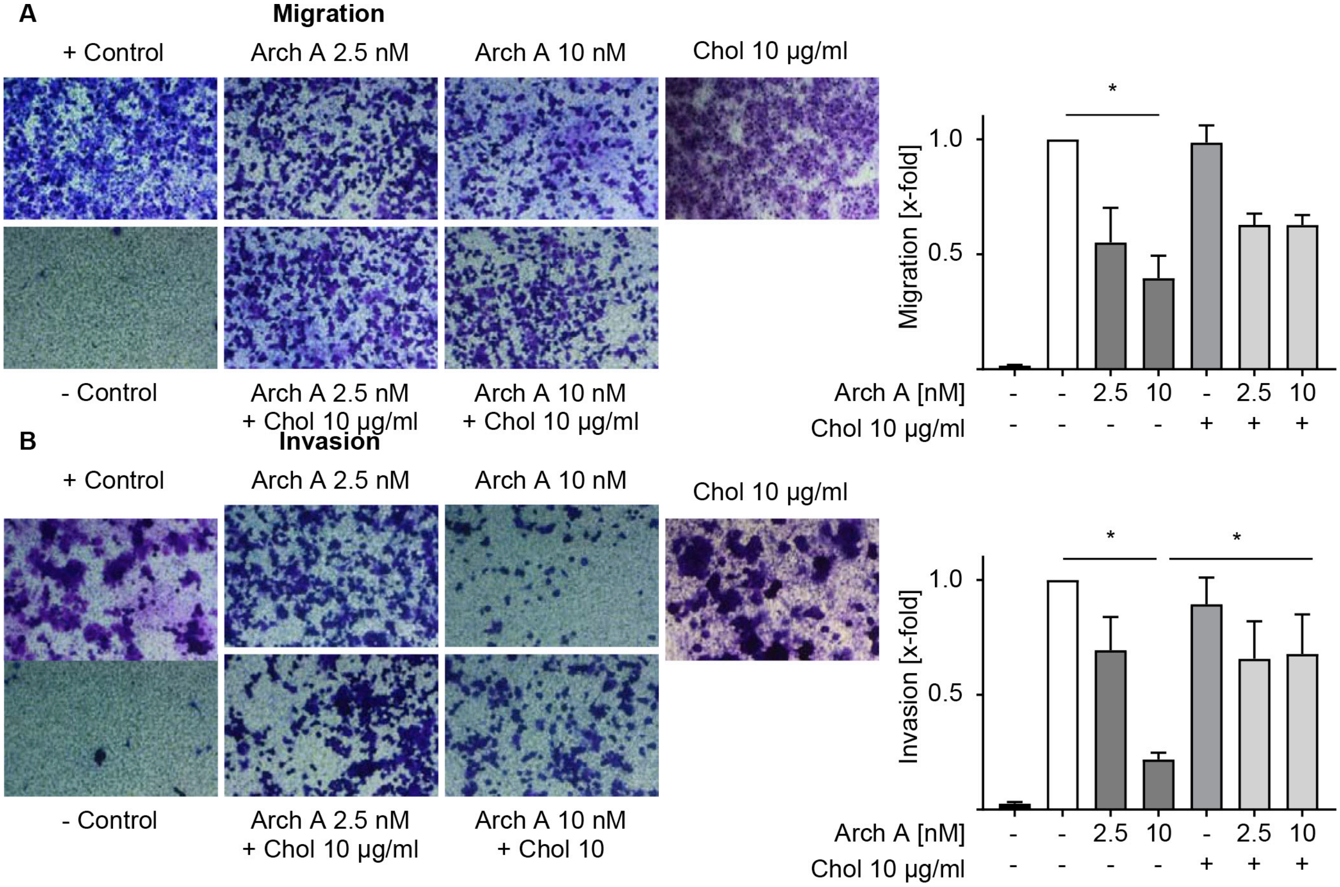

Supplementary Figure S4: Arch A influences migration and invasion of HCC cells HUH-7 cells were treated as indicated for 24 h, and allowed to migrate (**A**) in Transwell® chambers for 16 h or to invade into Matrigel containing Transwell® chambers for 48 h (**B**). Representative images of three independent experiments are shown. Respective bars represent the the quantification (SEM) of three independent experiments. *$p < 0.05$ (One-way ANOVA, Dunnett post test).

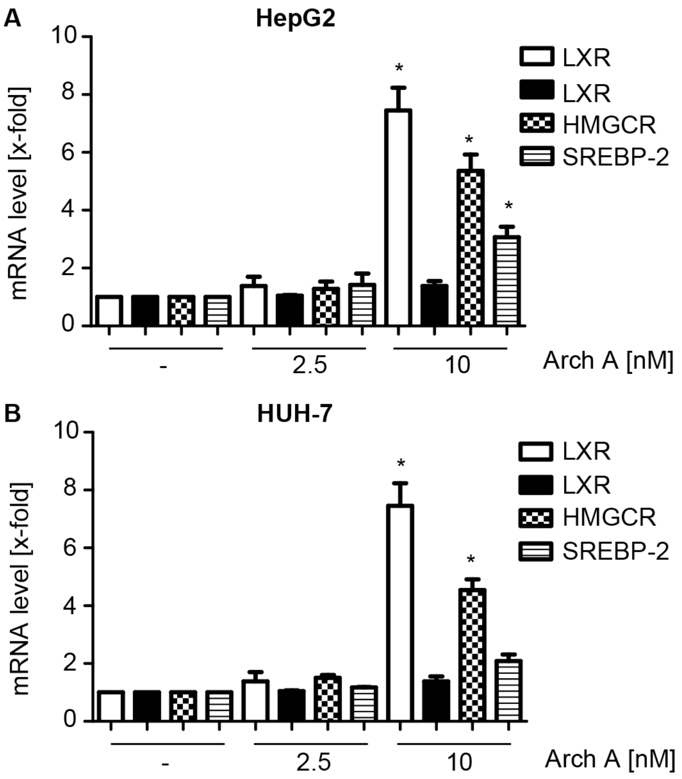

Supplementary Figure S5: Arch A influences expression levels of chol regulating genes. HUH-7 (**A**) and HepG2 (**B**) cells were treated as indicated with arch A (24 h) and relative mRNA expression levels of LDL-R, LXR, HMGCR and SREBP-2 were detected with the AB 7300 RealTime PCR system. Bars are the SEM of three independent experiments. *$p < 0.05$ (Repeated measures ANOVA, Dunnett post test).
